# Association of Bedtime with both Suicidal Ideation and Suicide Planning among Korean Adolescents

**DOI:** 10.3390/ijerph16203817

**Published:** 2019-10-10

**Authors:** Wonjeong Jeong, Yun Kyung Kim, Hyeon Ji Lee, Jieun Jang, Selin Kim, Eun-Cheol Park, Sung-In Jang

**Affiliations:** 1 Department of Public Health, Graduate School, Yonsei University, Seoul 03722, Korea; wjjeong@yuhs.ac (W.J.); hykh51128@yuhs.ac (Y.K.K.); leehj612@yuhs.ac (H.J.L.); jieun99@yuhs.ac (J.J.); lin3723@yuhs.ac (S.K.); 2 Institute of Health Services Research, Yonsei University, Seoul 03722, Korea; ecpark@yuhs.ac; 3 Department of Preventive Medicine, Yonsei University College of Medicine, Seoul 03722, Korea

**Keywords:** sleep, bedtime, suicidal ideation, suicide planning

## Abstract

Study Objectives: In comparison to other countries, the South Korean population has a short average sleep duration, and studies have suggested that insufficient sleep is a risk factor for suicidal behavior. This study aimed to examine the association of bedtime with suicidal ideation and with suicide planning, respectively, among Korean adolescents. Methods: This study included 48,218 participants from the 2017 Korea Youth Risk Behavior Web-based Survey. Participants were divided into three categories: pre-23:00 bedtime, 23:00–01:30 bedtime, and post-01:30 bedtime. Suicidal ideation and suicide planning were the main dependent variables, and multiple logistic regression analysis was performed to examine the target association. Results: For both male and female respondents, compared to those who had a pre-23:00 bedtime, those whose bedtime was after 01:30 were more likely to have suicidal ideation (post-01:30 bedtime for men: OR = 1.29, 95% CI = 1.16–1.45; for women: OR = 1.32, 95% CI = 1.20–1.44). For suicide planning, the results were also significant for both genders (post-01:30 bedtime for men: OR = 1.41, 95% CI = 1.16–1.70; for women: OR = 1.21, 95% CI = 1.03–1.43). Odds of suicidal ideation were higher for those who had a post-01:30 bedtime on weekdays but not weekends. Conclusions: We found that, among adolescents, going to bed after 01:30 is significantly associated with suicidal ideation and suicide planning, after adjusting for demographic, socioeconomic, and health-related characteristics. Therefore, late bedtime should be the timepoint of a suicide intervention for adolescents, in order to prevent developing suicidal ideations and suicide planning.

## 1. Introduction

The World Health Organization has stated that suicide is a serious public health problem, and that its prevention requires urgent attention [1]. Adolescent suicide is also the leading cause of death in the world and requires urgent prevention [2,3]. In 2018, South Korea had the second highest suicide rate among countries in the Organization for Economic Cooperation and Development (OECD) [4]; further, suicide was the cause of 28.4% of deaths among Korean adolescents in 2013, making it the primary cause of death among this age group [5]. The premature deaths of adolescents can have serious emotional effects for family members, and friends and, from a national perspective, can also negatively impact productivity [6,7]; thus, preventing adolescent suicide is a top priority for health and welfare [8].

Many factors can influence suicide, such as sociocultural, environmental, psychological, and biological elements [9,10,11]. Previous studies have highlighted several risk factors for suicidal ideation and suicidal behavior among adolescents, including peer victimization and psychiatric problems such as depression and anxiety [12]. In particular, sleep has long been identified as a factor that contributes to mental disorders [13], with several studies reporting that sleep disturbances such as insomnia, hypersomnia, and nightmares are risk factors for suicidal ideation and behaviors [10,13,14]. Insufficient sleep is associated with increased suicidal ideation and suicide attempts [13], and suicidal ideation and suicide attempts are also predictors of future suicidal behaviors [15]. Typically, suicide ideation is one of the first suicidal behaviors to manifest, which means intervening before the onset of suicide ideation is important [15]. Suicide planning is also associated with a high risk of suicide attempts [16]; one study reported that 60% of the transitions from suicidal ideation to suicide planning and suicide attempts occur within the first year of the onset of ideation [17]. Thus, in order to prevent suicide behavior, it is important to address suicidal ideation and suicide planning. To achieve this, information regarding suicide attempt risk factors is needed [16] and, considering the abovementioned findings, specific details regarding the effects of insufficient sleep.

Overall, the mean sleep duration across the 18 OECD countries is 502 min; however, in Korea the mean is just 469 min, which is the shortest duration among the surveyed nations [18]. Furthermore, although adolescent sleep health is becoming an internationally significant concern [19], Asian adolescents are more likely to go to bed late than are adolescents from western countries [20]. Regarding previous related examinations in the region, one study reported that suicidal ideation was more prevalent among Korean temporary workers who had less than six hours of sleep a night than among peers who had more than six hours of sleep a night [21]. Therefore, considering these observations and the high prevalence of suicide among Korean adolescents, it is clearly important to consider whether sleep duration is contributing to this issue. Moreover, as adolescents have fixed waking times as a result of school-related obligations, determining their bedtimes is also important in this regard.

Considering the above, it is necessary to investigate the association of bedtime with suicidal ideation and suicide planning, respectively, and to obtain evidence that could help prevent adolescents from maintaining late bedtimes, which could be a means of protecting youths from suicide ideation and suicide planning. Consequently, the current study sought to examine the association of bedtime with suicidal ideation and suicide planning, respectively, among Korean adolescents. We hypothesized that suicide ideation and suicide planning would be higher in adolescents who have a post-01:30 bedtime than among those who have a pre-23:00 bedtime, after adjusting for demographic, socioeconomic, and health-related characteristics.

## 2. Materials and Methods

### 2.1. Data and Study Participants

Study data were obtained from the 13th Korea Youth Risk Behavior Web-based Survey (KYRBWS), which was conducted in 2017 by the Korea Centers for Disease Control and Prevention (KCDC). The KYRBWS was approved by the KCDC Institutional Review Board (2014-06EXP-02-P-A) in 2014. From 2015, the ethics approval for the KYRBWS was waived by the KCDC Institutional Review Board under the Bioethics & Safety Act and opened to the public. All participants provided informed consent to participate in the KYRBWS and were guaranteed anonymity. The KYRBWS was an anonymous, Internet-based, self-administered structured questionnaire that used a complex research design, including multistage sampling, stratification, and clustering [22]. The survey comprised 123 questions assessing 15 areas of health-related behaviors. The survey’s target population was students in grades 7 through 12 in South Korea. For each grade level, sample classes were chosen at random from schools across the country, with all students in the selected classes being chosen as the sample students [22,23].

The total number of participants in the 2017 KYRBWS was 62,276 students. We excluded those who did not provide information regarding their age, allowance, economic status, grade, smoking status, alcohol consumption, intensity of physical activity, self-reported health status, and problematic mobile phone use. Consequently, a total of 48,218 participants (men: 23,391, women: 24,827) were included in this study sample.

### 2.2. Variables

The main independent variable was bedtime. The respondents in our study were asked to indicate the average time at which they went to bed on a weekday (Monday to Friday) and on a weekend (Saturday and Sunday). We then calculated the average time for weekdays and weekends. For the ‘bedtime’ variables, hours were not counted in terms of a.m. and p.m., but as 0 to 24 h after midnight were counted as follows: 25 for 1:00 a.m., 25.5 for 1:30 a.m., 26 for 2 a.m., etc. [24]. We then classified bedtime into three groups: pre-23:00, 23:00–01:30, and post-01:30. Additionally, the analysis included demographic, socioeconomic, and health-related characteristics. The demographic variables comprised age and gender, while the socioeconomic variables consisted of allowance, economic status, and grade. Health-related characteristics were smoking status, alcohol consumption, intensity of physical activity, self-reported health status, and problematic mobile phone use. Gender was compared within the gender, because gender could be affected by biological and psychological differences. The mobile phone use variables included whether respondents’ mobile phone use negatively impacted their relationships with their family and/or friends and also whether their mobile phone use interfered with their study.

Suicidal ideation and suicide planning were the main dependent variables in this study. That is, self-reported data regarding suicidal ideation were obtained from responses to the question: “have you felt a desire to kill yourself during the past year?” Suicidal ideation was categorized based on whether respondents answered “yes” or “no”. Meanwhile, suicide planning was determined based on responses to the question: “have you made a plan to kill yourself during the past year?” As above, suicide planning was determined based on whether respondents answered “yes” or “no”.

### 2.3. Statistical Analysis

Chi-square tests were used to investigate the general characteristics of the study population (Table S1). Meanwhile, multiple logistic regression analysis was performed to examine the association of bedtime with suicidal ideation and suicide planning, respectively, after accounting for potential confounders, including demographic, socio-economic, and health-related characteristics. The results were reported as odds ratios (OR) and 95% confidence intervals (CI). Subgroup analyses were also performed, using multiple logistic regression that was stratified in terms of problematic mobile phone use, grade, self-reported health status, and physical activity. The analysis used the stratified sampling variable (strata) and clustering variable (cluster) provided by the KYRBWS. P-values of <0.05 were considered to indicate statistically significant differences. All analyses were performed using SAS 9.4 software.

## 3. Results

Table S1 presents the general characteristics of the study population. Among the participants (23,391 men and 24,827 women), 2081 men (8.9%) and 3698 women (14.9%) showed suicidal ideation. Meanwhile, 681 men (2.9%) and 1020 women (4.1%) showed suicide planning. Bedtime showed a statistically significant relationship with both suicidal ideation and suicide planning.

Table 1 shows the association of bedtime with suicidal ideation and suicide planning, respectively. Compared to respondents with a pre-23:00 bedtime, those with a post-01:30 bedtime were more likely to show suicidal ideation and suicide planning. These results were significant for both men and women (suicidal ideation—men with a post-01:30 bedtime: OR = 1.29,95% CI = 1.16–1.45; women with a post-01:30 bedtime: OR = 1.32, 95% CI = 1.20–1.44; suicide planning—men with a post-01:30 bedtime: OR = 1.41, 95% CI = 1.16–1.70; women with a post-01:30 bedtime: OR = 1.21, 95% CI = 1.03–1.43). Furthermore, for both men and women, those who smoked and/or drank alcohol were more likely to show suicidal ideation (smoking status—men: OR = 1.47, 95% CI = 1.32–1.65; women: OR = 1.65, 95% CI = 1.43–1.89; drinking status—men: OR = 1.44, 95% CI = 1.29–1.60; women: OR = 1.64, 95% CI = 1.51–1.78) and suicide planning (smoking status—men: OR = 1.41, 95% CI = 1.16–1.71; women: OR = 1.94, 95% CI = 1.59–2.36; drinking status—men: OR = 1.63, 95% CI = 1.36–1.97; women: OR = 1.82, 95% CI = 1.57–2.11). For both men and women, as self-reported health status increased, the likelihood of suicidal ideation and suicide planning reduced (*p* < 0.05). Finally, those who reported problematic mobile phone use were more likely to show suicidal ideation and suicide planning (suicidal ideation—men: OR = 1.52, 95% CI = 1.38–1.69; women: OR = 1.86, 95% CI = 1.69–2.05; suicide planning—men: OR = 1.05, 95% CI = 0.89–1.24; women: OR = 1.45, 95% CI = 1.21–1.74).

**Figure 1:**
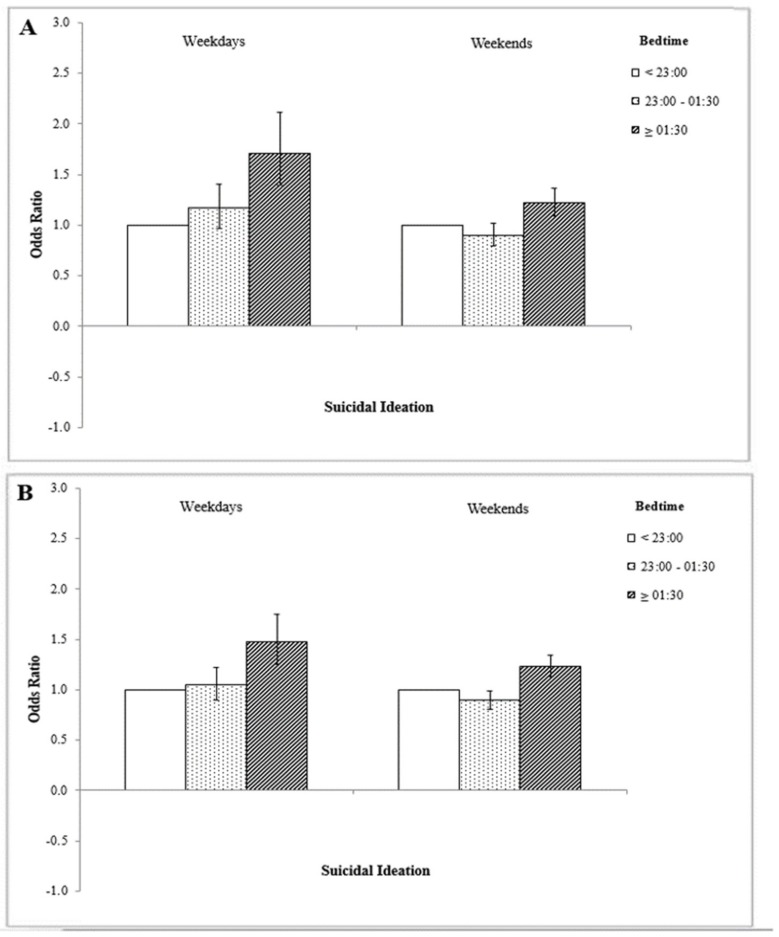
(**A**) Difference in suicidal ideation among men in terms of bedtimes on weekdays and weekends. (**B**) Difference in suicidal ideation among women in terms of bedtimes on weekdays and weekends. Adjusted for age, allowance, economic status, grade, smoking status, alcohol status, physical activity, self-reported health status, and mobile phone addiction variables. Reference group is ‘pre-23:00 bedtime’.

Figure 1 shows the association between bedtime on weekdays and weekends, respectively, and suicidal ideation in terms of gender. For both men and women, compared to respondents with a post-01:30 bedtime on weekends, those with a post-01:30 bedtime on weekdays were more likely to have suicidal ideation (*p* < 0.05).

**Figure 2:**
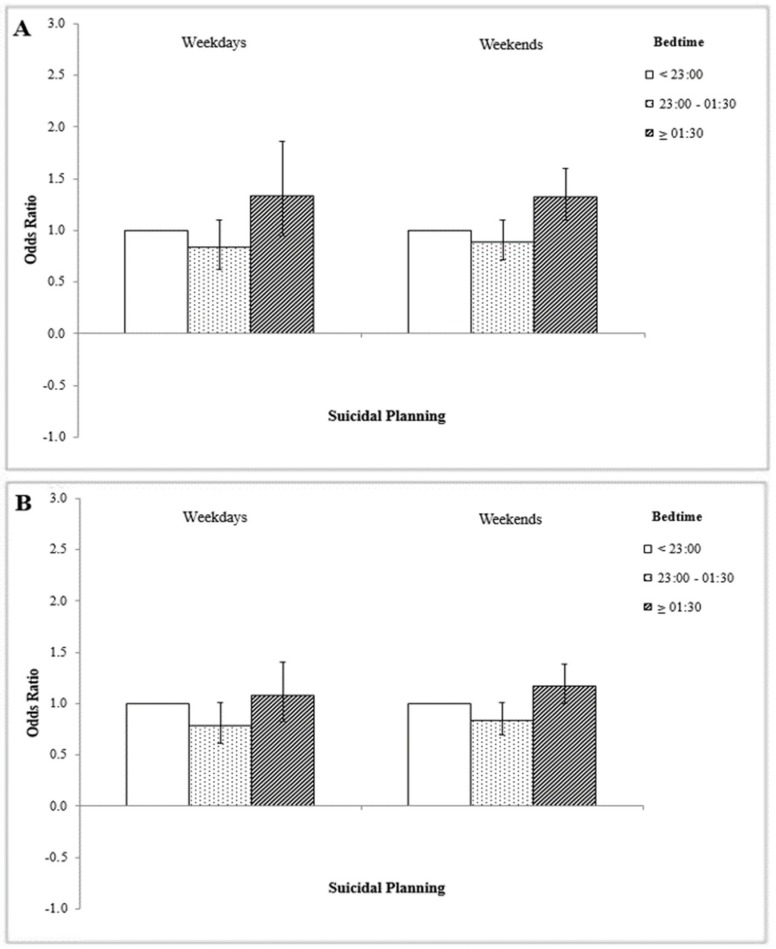
(**A**) Difference in suicide planning among men in terms of bedtime on weekdays and weekends. (**B**) Difference in suicide planning among women in terms of bedtime on weekdays and weekends. Adjusted for age, allowance, economic status, grade, smoking status, alcohol status, physical activity, self-reported health status, and mobile phone addiction variables. Reference group is ‘pre-23:00 bedtime’.

Figure 2 shows the association between bedtime on weekdays and weekends, respectively, and suicide planning in terms of gender. For both men and women, there was no significant difference in this regard between people with post-01:30 bedtimes on weekends and those with a post-01:30 bedtime on weekdays.

Table 2 and Table 3 shows results of subgroup analyses for the association of bedtime with suicidal ideation and suicide planning, respectively, stratified in terms of problematic mobile phone use, grade, self-reported health status, and physical activity. Regarding problematic mobile phone use, male respondents with a post-01:30 bedtime had higher odds of showing suicidal ideation and suicide planning than did those with a pre-23:00 bedtime (suicidal ideation: OR = 1.31, 95% CI = 1.15–1.49; suicide planning: OR = 1.42, 95% CI = 1.13–1.78). With respect to grade, adolescents with a post-01:30 bedtime were more likely to show suicidal ideation and suicide planning than were those with a pre-23:00 bedtime. However, the odds slightly increased for males who had high grades and who had a post-01:30 bedtime (suicidal ideation—high grades: OR = 1.33, 95% CI = 1.10–1.61; suicide planning—high grades: OR = 1.62, 95% CI = 1.18–2.22). In terms of health-related variables, respondents who had a post-01:30 bedtime and who had tendencies such as lower self-reported health status and lower physical activity were more likely to show suicidal ideation and suicide planning. However, the odds were also high for those with a post-01:30 bedtime and who had high self-reported health status and who engaged in a high level of physical activity.

## 4. Discussion

The purpose of this study was to examine the association of bedtime with suicidal ideation and suicide planning, respectively, among Korean adolescents. Through our analysis, we obtained evidence that earlier bedtimes for adolescents may contribute to preventing suicide ideation and suicide planning among such individuals. Our findings suggest that, among Korean adolescents, a post-01:30 bedtime has a significant relationship with suicidal ideation and suicide planning when compared with a pre-23:00 bedtime. These results indicate that going to bed before 23:00 may help to prevent the development of suicidal ideation and suicide planning.

One of the reasons adolescents go to bed late is that they are using the Internet and/or mobile phones late at night [25,26]. Adolescent addiction to the Internet through their mobile phones is becoming a serious problem [25,27]. A previous study reported that extensive Internet use creates a heightened level of psychological arousal, resulting in reduced sleep and mental health problems such as depression and anxiety [28]. Similarly, our study showed that mobile phone use, including conflicts with parents or friends as a result of mobile phone use, has an association with suicidal ideation and suicide planning, especially among individuals who have a post-01:30 bedtime (when compared to those with a pre-23:00 bedtime). Mobile phone addiction can affect both sleep quality and psychological health, and could consequently predict suicidal ideation and suicide planning [25]. Moreover, our study also shows that those who did not report having a problematic mobile phone use, were also associated with suicidal ideation and suicide planning among individuals who have a post-01:30 bedtime (when compared to those with a pre-23:00 bedtime). A previous study reported that mobile phone use after lights out may be associated with poor mental health, and suicidal feeling for adolescents. This shows that nocturnal mobile phone use could worsen their mental health [29]. Therefore, in order to prevent developing suicidal ideation and suicide planning, dealing with adolescents’ mobile phone use at night could be important.

Regarding grades, we found that individuals who have a post-01:30 bedtime are more likely to have suicidal ideation and suicide planning than are those who have a pre-23:00 bedtime. Interestingly, grade level had little effect in this regard; only suicide planning among men increased with higher grades. A previous study reported that academic performance among adolescents induces insufficient sleep syndrome; many adolescents believe that academic performance is important for preparing themselves for adulthood, and most of the adolescents examined in this previous study reported experiencing stress regarding their grades, regardless of whether their grades were high or low [30]. Furthermore, another study stated that adolescents in South Korea have shorter sleep durations than do adolescents in other countries, and that academic performance influences this trend [30,31].

Regarding health-related variables, individuals who had a post-01:30 bedtime were determined to be more likely to show suicidal ideation and suicide planning than were those who had a pre-23:00 bedtime. This shows that, regardless of physical health, going to bed late negatively influences suicidal ideation and suicide planning [13].

Interestingly, the odds of developing suicidal ideation were lower among individuals who had a pre-01:30 bedtime on weekends than among those who went to bed at this time on weekdays. This shows that adolescents who usually go to bed late on weekdays because of worries regarding academic performance in school could have higher suicidal ideation [30]. However, for suicide planning, there was no significant difference between those who had a pre-01:30 bedtime on weekends only and those who went to bed at this time on weekdays. As, compared to suicidal ideation, suicide planning is associated with a higher risk of attempted suicide [16], this indicates that going to bed late on weekends can also impact on development of suicide planning.

It should be noted that the current study has several limitations. First, the results of this study are based on self-report responses. As a result, some survey questions may have been subject to recall bias, especially those regarding health-related characteristics. Moreover, responses could also have been affected by social desirability bias. Thus, caution should be taken when interpreting the results. Second, as a result of this study’s cross-sectional design, cause and effect, as well as the direction of the relationships observed, cannot be determined. Third, our measures of suicidal ideation, suicide planning, and bedtime were based on single questions in the survey, which were not specially designed to examine the association of bedtime with suicidal ideation and suicide planning, respectively. Moreover, as there is a tendency for respondents to show reluctance regarding answering questions related to suicide, there might have been more people in the sample with suicidal ideation and suicide planning. However, even if the number in this regard increased, the effect would likely remain significant.

Despite these limitations, our study has numerous strengths. The KYRBWS survey is representative of the overall adolescent student population in Korea in grades 7 through 12. The survey used a complex design that included multistage sampling, stratification, and clustering. Therefore, the results can be generalized to the overall adolescent population in South Korea. Furthermore, as the KYRBWS was a web-based anonymous survey, obtaining honest responses was relatively likely. Moreover, the results from the current study can be used as a baseline for motivating adolescents to go to bed early and for protecting them from suicidal ideation and suicide planning.

## 5. Conclusions

The current study identified a significant relationship between bedtime and suicidal ideation and suicide planning, respectively. Our findings suggest that individuals who have a post-01:30 bedtime are more likely to show suicidal ideation and suicide planning than are those who have a pre-23:00 bedtime. As 60% of the transitions from suicidal ideation to suicide planning and suicide attempt occur within the first year of the onset of ideation [17], it is important to prevent suicidal ideation and suicide planning. Taken together, by highlighting the association of bedtime with suicidal ideation and suicide planning, respectively, the results of the current study could motivate parents and/or teachers to monitor adolescents who go to bed late at night. Moreover, as a previous study shows, considering delaying school start time could benefit adolescents from sleep duration and mental health [32]. In particular, this research suggests that more support and attention should be devoted to adolescents, especially those who go to bed late.

## Figures and Tables

**Table 1:** Factors associated with suicidal ideation and suicide planning

Variables		Male				Female			
		Suicidal Ideation		Suicide Planning		Suicidal Ideation		Suicide Planning	
		Adjusted OR	95% CI	Adjusted OR	95% CI	Adjusted OR	95% CI	Adjusted OR	95% CI
Bedtime	<23:00	1.00		1.00		1.00		1.00	
	23:00–01:30	0.97	(0.86–1.10)	0.90	(0.74–1.10)	0.98	(0.89–1.08)	0.96	(0.81–1.15)
	≥01:30	1.29	(1.16–1.45)	1.41	(1.16–1.70)	1.32	(1.20–1.44)	1.21	(1.03–1.43)
Age	12–15	1.29	(1.15–1.44)	1.66	(1.40–1.97)	1.69	(1.55–1.85)	2.17	(1.85–2.55)
	16–18	1.00		1.00		1.00		1.00	
Allowance	Low	1.09	(0.94–1.26)	1.01	(0.80–1.28)	1.09	(0.98–1.22)	1.11	(0.91–1.35)
	Lower Middle	0.98	(0.86–1.13)	0.84	(0.67–1.05)	0.94	(0.84–1.05)	0.93	(0.77–1.13)
	Upper Middle	0.95	(0.82–1.09)	0.94	(0.75–1.17)	0.96	(0.86–1.07)	0.86	(0.71–1.04)
	High	1.00		1.00		1.00		1.00	
Economic Status	Low	1.43	(1.25–1.63)	1.19	(0.96–1.48)	1.34	(1.19–1.50)	1.38	(1.15–1.65)
	Middle	0.83	(0.75–0.92)	0.73	(0.61–0.88)	0.89	(0.82–0.97)	0.83	(0.72–0.97)
	High	1.00		1.00		1.00		1.00	
Grade	High	0.79	(0.71–0.89)	0.75	(0.62–0.91)	0.80	(0.73–0.87)	0.78	(0.67–0.91)
	Middle	0.84	(0.75–0.95)	0.87	(0.72–1.06)	0.80	(0.73–0.88)	0.77	(0.65–0.91)
	Low	1.00		1.00		1.00		1.00	
Smoking Status	Never	1.00		1.00		1.00		1.00	
	Ever	1.47	(1.32–1.65)	1.41	(1.16–1.71)	1.65	(1.43–1.89)	1.94	(1.59–2.36)
Alcohol Status	Never	1.00		1.00		1.00		1.00	
	Ever	1.44	(1.29–1.60)	1.63	(1.36–1.97)	1.64	(1.51–1.78)	1.82	(1.57–2.11)
Physical Activity	Low	0.97	(0.88–1.06)	0.91	(0.77–1.07)	0.77	(0.70–0.84)	0.68	(0.59–0.79)
	High	1.00		1.00		1.00		1.00	
Self-Reported Health Status	High	0.24	(0.20–0.28)	0.25	(0.20–0.32)	0.24	(0.21–0.27)	0.22	(0.19–0.27)
	Middle	0.52	(0.44–0.62)	0.52	(0.40–0.68)	0.49	(0.44–0.55)	0.51	(0.42–0.61)
	Low	1.00		1.00		1.00		1.00	
Problematic Mobile Phone Use	Yes	1.52	(1.38–1.69)	1.05	(0.89–1.24)	1.86	(1.69–2.05)	1.45	(1.21–1.74)
	No	1.00		1.00		1.00		1.00	

**Table 2:** Subgroup analysis of the association of bedtime with suicidal ideation stratified by covariates

Variables		Suicidal Ideation				
		<23:00	23:00–01:30		≥01:30	
		Adjusted OR	Adjusted OR	95% CI	Adjusted OR	95% CI
Male						
Problematic Mobile Phone Use	Yes	1.00	1.03	(0.89–1.19)	1.31	(1.15–1.49)
	No	1.00	0.84	(0.67–1.06)	1.25	(1.00–1.56)
Grade	High	1.00	1.00	(0.82–1.22)	1.33	(1.10–1.61)
	Middle	1.00	0.99	(0.78–1.26)	1.26	(1.01–1.57)
	Low	1.00	0.94	(0.77–1.16)	1.30	(1.06–1.58)
Self-Reported Health Status	High	1.00	0.95	(0.82–1.11)	1.34	(1.15–1.56)
	Middle	1.00	0.92	(0.73–1.15)	1.21	(0.97–1.50)
	Low	1.00	1.29	(0.89–1.86)	1.31	(0.93–1.86)
Physical Activity	Low	1.00	0.92	(0.78–1.08)	1.26	(1.07–1.48)
	High	1.00	1.03	(0.87–1.22)	1.32	(1.11–1.56)
Female						
Problematic Mobile Phone Use	Yes	1.00	0.98	(0.89–1.09)	1.27	(1.15–1.40)
	No	1.00	0.99	(0.79–1.24)	1.57	(1.27–1.94)
Grade	High	1.00	0.93	(0.80–1.10)	1.32	(1.13–1.53)
	Middle	1.00	0.93	(0.77–1.12)	1.31	(1.08–1.57)
	Low	1.00	1.07	(0.91–1.26)	1.32	(1.13–1.53)
Self-Reported Health Status	High	1.00	0.97	(0.85–1.10)	1.28	(1.13–1.45)
	Middle	1.00	0.92	(0.77–1.10)	1.29	(1.10–1.51)
	Low	1.00	1.26	(0.92–1.71)	1.54	(1.20–1.99)
Physical Activity	Low	1.00	0.97	(0.86–1.09)	1.32	(1.19–1.48)
	High	1.00	1.01	(0.85–1.20)	1.29	(1.08–1.52)

**Table 3:** Subgroup analysis of the association of bedtime with suicide planning, stratified by covariates

Variables		Suicide Planning				
		<23:00	23:00–01:30		≥01:30	
		Adjusted OR	Adjusted OR	95% CI	Adjusted OR	95% CI
Male						
Problematic Mobile Phone Use	Yes	1.00	0.88	(0.69–1.14)	1.42	(1.13–1.78)
	No	1.00	0.92	(0.65–1.29)	1.39	(0.99–1.94)
Grade	High	1.00	0.93	(0.66–1.31)	1.62	(1.18–2.22)
	Middle	1.00	0.83	(0.55–1.27)	1.53	(1.06–2.21)
	Low	1.00	0.94	(0.67–1.33)	1.18	(0.86–1.62)
Self-Reported Health Status	High	1.00	0.83	(0.64–1.08)	1.41	(1.10–1.82)
	Middle	1.00	0.94	(0.63–1.40)	1.10	(0.76–1.59)
	Low	1.00	1.31	(0.71–2.44)	2.38	(1.33–4.26)
Physical Activity	Low	1.00	0.80	(0.59–1.07)	1.15	(0.88–1.50)
	High	1.00	1.00	(0.76–1.33)	1.71	(1.29–2.28)
Female						
Problematic Mobile Phone Use	Yes	1.00	0.98	(0.81–1.19)	1.13	(0.94–1.35)
	No	1.00	0.89	(0.60–1.30)	1.70	(1.18–2.43)
Grade	High	1.00	0.88	(0.67–1.16)	1.19	(0.91–1.57)
	Middle	1.00	0.89	(0.63–1.26)	1.17	(0.85–1.62)
	Low	1.00	1.10	(0.82–1.47)	1.27	(0.97–1.67)
Self-Reported Health Status	High	1.00	0.92	(0.73–1.16)	1.24	(0.96–1.59)
	Middle	1.00	1.02	(0.75–1.38)	1.24	(0.94–1.63)
	Low	1.00	1.01	(0.65–1.58)	1.15	(0.80–1.65)
Physical Activity	Low	1.00	0.99	(0.80–1.22)	1.25	(1.02–1.53)
	High	1.00	0.90	(0.66–1.23)	1.13	(0.85–1.52)

## Supplementary Materials

The following are available online at https://www.mdpi.com/1660-4601/16/20/3817/s1, Table S1: General characteristics of the study population.
